# Cost of Ambulatory Care in Diabetes: Findings From a Non-Communicable Disease Clinic of a Tertiary Care Institute in Eastern India

**DOI:** 10.7759/cureus.21206

**Published:** 2022-01-13

**Authors:** Binod K Patro, Manish Taywade, Debjyoti Mohapatra, Rashmi R Mohanty, Kishore K Behera, Soumya S Sahoo

**Affiliations:** 1 Community Medicine and Family Medicine, All India Institute of Medical Sciences, Bhubaneswar, Bhubaneswar, IND; 2 Community Medicine, Srirama Chandra Bhanja Medical College, Cuttack, IND; 3 General Medicine, All India Institute of Medical Sciences, Bhubaneswar, Bhubaneswar, IND; 4 Endocrinology, All India Institute of Medical Sciences, Bhubaneswar, Bhubaneswar, IND; 5 Community and Family Medicine, All India Institute of Medical Sciences, Bathinda, Bathinda, IND

**Keywords:** india, ambulatory care, cost of illness, primary care, diabetes

## Abstract

Background: This study was conducted to evaluate the cost of ambulatory care of diabetes in a non-communicable disease (NCD) clinic in eastern India.

Methods: This hospital-based cross-sectional cost description study was conducted from July to August 2018. A total of 192 diagnosed cases aged 18-70 years with a minimum history of one year since diagnosis attending the NCD clinic for the first time were included. Information was collected using a pre-tested schedule based on the cost of illness approach that consisted of socio-demographic details, disease status, and cost of ambulatory care. Cost of the drugs was calculated using a standardized repository of drug costs. The estimated expenditure of previous three months was calculated and extrapolated to one year to calculate yearly expenditure.

Results: The mean age of the study participants was 43.93±10.41 years and the mean duration of diabetes was 6.64±6.08 years. The median direct cost due to diabetes was Rs 9560 (136.57 USD) annually. It was higher in females (Rs 10,056, 143.45 USD) than in males (Rs 9020, 128.85 USD). In direct medical costs, a major part was constituted by the drugs, oral hypoglycemic agents, and/or insulin (approximately 70%).

Conclusions: In an ambulatory framework too, diabetes causes a substantial financial burden on the individual in India. In the wake of resource constraints in Indian health settings, the public health system needs to be adequately strengthened by policymakers to address the growing number of diabetics and long-standing complications.

## Introduction

India is experiencing a growing burden of non-communicable diseases (NCDs). The disease landscape has vastly transitioned from infectious diseases to a predominantly chronic NCD pattern. Unfortunately, the pace of growth of NCDs in India has been alarmingly high. They contribute to around 41 million (70%) of all the deaths globally and about 5.81 million (61%) of all deaths in India [1]. Four NCDs primarily responsible for mortality and morbidity are cardiovascular diseases, chronic respiratory disease, cancers, and diabetes, contributing to about 82% of all NCD deaths [2]. India is regarded as the diabetes capital of the world, entirely because of the number of rising new cases and a large burden of old prevalent cases. The Indian Council of Medical Research-India Diabetes (ICMR- INDIAB) study in 15 states of India showed the overall prevalence of diabetes to be 7.3% [3]. The Global Burden of Disease (GBD) 2016 study reported diabetes contributes 3.1% (95% CI 2.9-3.3) of the total mortality burden, with slightly higher contributions among women than men. The age-standardized diabetes prevalence rose by 29.7% from 1990 to 2016 [4]. In absolute numbers, the prevalence of diabetes is projected to increase to 80 million by 2030, placing an immense burden on healthcare resources [5].

Exacerbating this problem are the accompanying multiple chronic conditions and the fact that many remain undiagnosed, due to lack of awareness and insufficient healthcare access [6]. These diseases because of their chronic nature and high burden of morbidity need a long-term continuum of care. Though there has been a significant increase in the burden of diabetes and other NCDs in recent times, there is a lack of integration of primary healthcare with the programmatic implementation of NCD management in India. Diabetes, being a long-standing disease with complications, makes it challenging for the underprepared health systems to deal with the frequent and intensive encounters with the disease [7]. Along with the long course of the disease, the financial implications of the frequent costs, consultations, medications, and different indirect costs put a significant economic burden on the people. The Lancet global cost of illness study for diabetes estimated that the global cost of diabetes for 2015 was 1.31 trillion USD or 1.8% of the global gross domestic product (GDP). It also highlighted the substantial variations in the share and composition of indirect costs across countries [8]. There has been scarce literature on studies regarding the comprehensive cost of diabetes from developing countries, with relatively few of them being from India. In this context, as most of the patients with diabetes are treated as outpatients, we conducted this ambulatory cost of care for diabetes study in an NCD clinic of a tertiary care institute in eastern India.

## Materials and methods

Study setting

This cross-sectional cost description study was conducted in the NCD clinic of a tertiary care center of eastern India that caters to the needs of entire Odisha and neighboring states like West Bengal. The NCD clinic is managed by community medicine physicians with a daily attendance of about 50 patients. The recruitment of study participants and data collection was done over a period of three months from June to August 2018. A total of 192 type 2 diabetic subjects were included in the study. All diagnosed cases of diabetes mellitus with a minimum history of six months since diagnosis and with age greater than 18 years attending the NCD clinic, of All India Institute of Medical Sciences, Bhubaneswar, for the first time were eligible for inclusion.

Study methodology and tool

**Figure 1:**
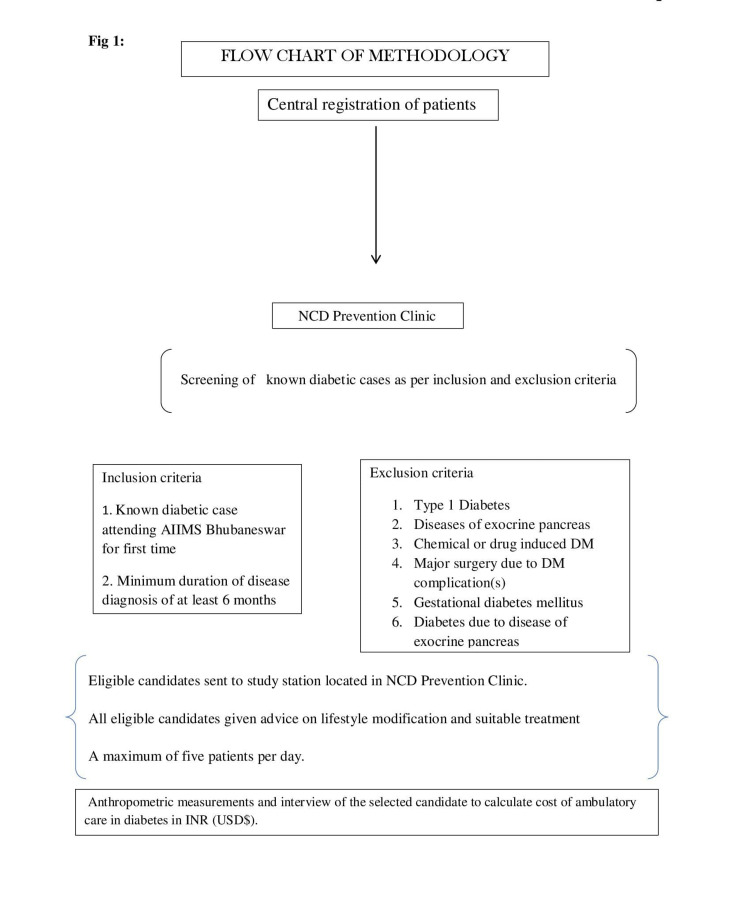
Flowchart of the methodology process NCD, non-communicable disease; AIIMS, All India Institute of Medical Sciences; DM, diabetes mellitus; INR, Indian rupee

Patients attending the NCD clinic were screened by the Senior Resident/Faculty of Community Medicine for eligibility. The maximum number of subjects to be enrolled in a day was limited to five to maintain the quality of data. Subjects found eligible were sent to the study station situated in the NCD clinic where they were interviewed by a trained person (medical social worker) (Figure 1). Data was collected using a pre-tested schedule based on the cost of illness approach that consisted of the following sections: (a) socio-economic and demographic detail of participants, (b) details of their disease status, (c) health-seeking behavior in the past three months, and (d) cost of ambulatory care in diabetes management including direct medical and non-medical costs.

Direct medical cost captured the cost of drugs related to diabetes and complications related to diabetes (hypertension, coronary artery disease, dyslipidemia, peripheral neuropathy, diabetic kidney disease, and diabetic retinopathy), cost of consultation, cost of investigation advised in relation to the disease, and cost of self-monitoring. Direct non-medical cost captured the data on the cost of transportation and cost of food. To keep recall bias to a minimum, only the expenditure incurred in the past three months was taken into account. The patients were asked to show the drugs taken during the past three months or their prescription. In case patients could not recall or produce the drugs or past prescriptions during the personal interview, they were asked by the interviewer to confirm the same telephonically once they returned home. Wherever possible, the image of the drug packets was shared with the interviewer using messaging apps. The cost of the drugs was calculated using a standardized repository of drug costs for medical cost and as per the existing prices on the day of data collection. The estimated expenditure of the previous three months was calculated and was extrapolated to one year to calculate the patient’s estimated yearly expenditure.

Data were entered into MS Excel and analysed using IBM SPSS Statistics for Windows, Version 20.0 (IBM Corp., Armonk, NY). The median cost of ambulatory care was expressed as annual cost in Indian rupees. The study was approved by the Institutional Ethics Committee of All India Institute of Medical Sciences, Bhubaneswar.

## Results

Socio-demographic profile

A total of 192 diabetic patients were included in our study. Out of them, more than two-thirds, 66.1 % (n=127), were males and a majority (78.1%) of the study participants were in the 30-59 age group. The mean age of the study participants was 43.93±10.41 years and the mean duration of diabetes was 6.64±6.08 years. The majority, 132 (68.8%), of them were residing in rural areas and nearly one-fifth, 38 (18.7%), of the study participants were unemployed or retired. Nearly one-third, 57 (29.7%), of them belonged to the below poverty line category (Table 1). The mean age at diagnosis was marginally higher in males (44.57±10.92 years) than females (43.51±9.59 years).

**Table 1:** Socio-demographic characteristic of participants APL, above poverty line; BPL, below poverty line

Characteristic	Number (%)
Gender	
Male	127 (66.1)
Female	65 (33.9)
Age (years)	
<30	2 (1.0)
30-59	150 (78.1)
≥60	40 (20.9)
Education	
Illiterate	11 (5.7)
Primary	50 (26.0)
Secondary	44 (22.9)
Middle	29 (15.1)
Matriculate	33 (17.2)
Graduate and above	25 (13.0)
Residence	
Rural	132 (68.8)
Urban	60 (30.8)
Current marital status	
Married	178 (92.7)
Unmarried	3 (1.6)
Widower	9 (4.7)
Separated	2 (1)
Occupation	
Unemployed	11 (5.6)
Homemaker	53 (27.6)
Govt service	25 (13.0)
Business/Self-employed	52 (27.1)
Semi/Unskilled	22 (11.5)
Retired	29 (15.1)
Monthly family income	
APL	135 (70.3)
BPL	57 (29.7)
Social group	
General	68 (35.4)
Other backward class	99 (51.6)
Schedule caste/schedule tribe	25 (13.0)

Care-seeking behavior

In the majority, 139 (72.4%), of patients, the health-seeking behavior for diabetes was self-initiated, when they first experienced the symptoms and were subsequently diagnosed. More than half of the participants had their first diagnosis in the private sector (private practitioner, nursing home, and hospitals). Three study subjects admitted to being diagnosed by a traditional faith healer/quack. However, in the last three months, more than half were seeking care/having checkups in government institutions. In terms of the consulting doctors, nearly half (51.2%) of the study subjects sought care from a general physician whereas about one-third (31.8%) of them sought care from either a specialist or super-specialist. Most (148, 77.1%) of the patients were on oral hypoglycemic agents (OHAs) while some of them were on insulin and OHAs both. It was worrying to see that 16 (8.3%) patients had not taken any medicines in the previous three months. Two-thirds of the patients had some form of long-term complications at the time of enrollment, microvascular or macrovascular or both. Hypertension was the most common associated co-morbidity. It was quite encouraging to see that nearly half of the patients had no co-morbidities associated (Table 2).

**Table 2:** Details of diabetes among study participants IQR, interquartile range; PHC, primary health center; CHC, community health center; DH, district hospital; OHA, oral hypoglycemic agent; CAD, coronary artery disease; TB, tuberculosis; COPD, chronic obstructive pulmonary disease

	Male (n=127) (%)	Female (n=65) (%)	Total (n=192) (%)
Age at first diagnosis (years)	44.57±10.92	43.51±9.59	43.93±10.41
Duration of diabetes (years); median (IQR)	4 (8)	3 (5)	4 (7)
Initiation of first diagnosis			
Self-initiated	95 (74.8)	44 (67.7)	139 (72.4)
Opportunistic/incidental	31 (24.4)	23 (30.8)	55 (26.6)
Others	1 (0.8)	1 (1.5)	2 (1.0)
Place of diagnosis			
Govt health facility	31 (24.4)	21 (32.3)	52 (27.1)
Private practitioner	54 (42.5)	25 (38.5)	83 (41.1)
Polyclinic	14 (11.0)	5 (7.7)	19 (9.9)
Corporate hospitals	7 (5.5)	3 (4.6)	10 (5.2)
Medical college	19 (15.0)	10 (15.4)	29 (15.1)
Quack/traditional faith healer	2 (1.6)	1 (1.5)	3 (1.6)
Place of care in the last three months			
Govt health facility (PHC, CHC, DH)	45 (34.1)	22 (34.3)	67 (34.2)
Private practitioner	25 (19.4)	22 (33.8)	46 (24.5)
Polyclinics	8 (6.3)	2 (3.1)	10 (5.2)
Corporate hospitals	6 (4.7)	1 (1.5)	7 (3.6)
Medical college	18 (14.2)	8 (12.3)	26 (13.5)
Quack/traditional faith healer	5 (3.9)	2 (3.1)	7 (3.6)
Dual consultation	1 (0.8)	1 (1.5)	2 (1.0)
No consultation	19 (15.0)	7 (10.8)	28 (13.5)
Primary treating physician in the last three months			
General physician	65 (51.2)	27 (41.5)	92 (47.9)
Specialist	29 (22.8)	24 (36.9)	53 (27.6)
Super-specialist	6 (4.7)	2 (3.0)	8 (4.2)
Alternative system of medicine	7 (5.5)	4 (6.2)	11 (5.7)
Consulted two different grades of physician	1 (0.8)	1 (1.5)	2 (1.0)
Did not seek care	19 (14.7)	7 (10.8)	26 (13.5)
Medicines in the past three months			
OHA	97 (75.2)	51 (78.5)	148 (77.1)
Insulin	6 (6.2)	0 (1.5)	6 (3.1)
Insulin and OHA	12 (9.4)	6 (9.2)	18 (9.4)
Alternative system of medicine	2 (1.6)	2 (3.1)	4 (2.1)
None	10 (7.9)	6 (9.2)	16 (8.3)
Place from where medicine was taken			
Govt supply	9 (7.1)	5 (7.7)	14 (7.3)
Chemist shop	106 (83.5)	53 (81.5)	159 (82.8)
Both govt supply and chemist shop	2 (1.6)	1 (1.5)	3 (1.6)
Not taken medicine in the past three months	10 (7.9)	6 (9.2)	16 (8.3)
Complication			
Microvascular	60 (47.2)	33 (50.8)	93 (48.4)
Macrovascular	7 (5.5)	2 (3.1)	7 (3.6)
Both	8 (6.3)	5 (7.7)	13 (6.8)
None	53 (40.9)	25 (38.5)	77 (40.1)
Co-morbidity			
Hypertension	40 (31.5)	20 (30.8)	60 (31.2)
CAD	2 (1.6)	0 (0)	2 (1.0)
TB	1 (0.8)	0 (0)	1 (1.0)
COPD	3 (2.4)	1 (1.5)	4 (2.1)
Thyroid disorder	3 (2.4)	3 (4.6)	6 (3.1)
Others	4 (3.1)	0 (0)	4 (2.1)
Two co-morbidities	4 (3.1)	2 (3.1)	6 (3.1)
More than 2 co-morbidities	2 (1.6)	0 (0)	2 (1.0)
None	67 (52.8)	31 (47.7)	98 (51.0)

Cost of diabetes care

The median direct cost due to diabetes was Rs 9560 (136.57 USD) annually. The median total direct cost was found to be higher in females (Rs 10,056, 143.45 USD) than in males (Rs 9020, 128.85 USD). The direct medical costs due to diabetes alone in three months were Rs 1520 (873-1728). When the cost of drugs for complications and co-morbidities were included, the total cost was marginally higher at Rs 1931 (933-3244) in the past three months (Table 3). In this case too, gender-wise the cost was higher in females as compared to males. In the direct medical costs, the major part was constituted of the drugs, OHA and/or insulin (approximately 70%). The costs of consultation, investigation, and self-monitoring constituted only a fractional cost of the total direct cost. The direct non-medical cost, which included transport cost and food costs mainly, was also minimal.

**Table 3:** Median cost of diabetes care distribution according to gender OHA, oral hypoglycemic agent; IQR, interquartile range

	Total (in rupees); median (IQR)	Cost in males (n=127) (In rupees); median (IQR)	Cost in females (n=65) (In rupees); median (IQR)
Direct medical cost			
OHA and/or Insulin	1059 (576-1955)	1000 (571-1565.5)	1126 (637.5-2407.5)
Cost of consultation	0 (0-400)	0 (0-300)	0 (0-350)
Cost of investigation	160 (0-300)	120 (0-280)	160 (0-300)
Cost of self-monitoring	0 (0-300)	0 (0-0)	0 (0-0)
Total direct medical cost (without complications)	1520 (873-1728)	1471 (844.5-2682.0)	1717.5 (937-3232)
Total direct medical cost (with complications and co-morbidities)	1931 (933-3244)	1914 (920-2978)	2134 (964.2-3668.5)
Direct non-medical cost			
Transport cost	100 (0-300)	90 (0-200)	70 (0-350)
Food cost	40 (40-150)	25 (0-110)	10 (0-120)
Total direct non-medical cost	120 (0-330)	140 (10-300)	117.5 (0-392.5)
Total direct cost (medical and non-medical)	2390 (1096-3664)	2255 (1140.5-3415)	2514 (1022.2-4025.5)
Total direct cost annually	9560 (136.57 USD)	9020 (128.85 USD)	10,056 (143.65 USD)

## Discussion

Care of diabetes is a challenge in many aspects in a developing country like India. The economic implications of diabetes need to be studied upon in view of the clinical, health system, and societal implications. First and foremost, the private healthcare sector is the major provider of diabetes care in India, and being a chronic disease, it has large repercussions on out-of-pocket (OOP) expenditure [9]. Secondly, by 2025 most diabetics would be in the 45- to 64-year age group threatening the economic productivity of the country and earning ability of individuals [10].

The highest economic burden caused by diabetes is the monetary value associated with disability and loss of life as a result of the disease itself and related chronic complications. Also, it is not surprising that within diabetic patients, the greatest financial burden is borne by the poorest quintile. The median private sector OOP hospitalization expenditure is four times higher than the public sector (p < 0.001) [11]. In our study, among the direct costs, the cost of medicine (OHA and/or insulin) formed the bulk. This has been the case in many other studies in India [6,12-14]. However, the Cost of Diabetes in India (CODI) study outlined that anti-diabetic drug costs accounted only for 17% of direct medical expenses on diabetes care [15]. A secondary National Sample Survey Organization (NSSO) data analysis by Tripathy and Prasad reported medicines accounted for 69% of public sector outpatient care [11]. In our study, this may be attributed to the fact that a large majority of patients were buying the medications from chemist shops/private vendors. The chronic nature of the disease along with the daily need for medicines puts a great burden on the patients. This is exacerbated in patients on insulin and particularly in patients with uncontrolled diabetes. This makes the need for ensuring the essential anti-diabetic drugs in the public sector all the more important. Also, the increased sensitization of patients to *Jan Aushadhi* stores and other similar subsidized ventures can significantly reduce this financial burden. The poor availability and affordability of essential diabetes medicines in many communities, especially those from low and middle-income countries (LMIC) like India, does not help the cause [16]. The Global Action Plan for the Prevention and Control of Noncommunicable Diseases 2013-2020 sets a voluntary target of 80% availability of affordable essential medicines, required to treat major NCDs in both public and private facilities by 2025 [17].

The median cost in rupees for consultation and self-monitoring was negligible. This can be because of relatively stable patients and most of the patients requiring simple blood tests like fasting and postprandial that do not cost much. Alternatively, this may be due to poor self-monitoring practices and inadequate health-seeking behavior with less frequent visits for consultation. The direct non-medical costs due to transportation and food were also minimal as most of the patients came from nearby localities making the visits equivalent to daycare.

The median direct cost due to diabetes was Rs 9560 (USD 136.57) annually that was lower as estimated in comparison to some studies. A systematic review on cost of illness studies in LMIC outlined a clear trend of increment in the average annual direct cost per person per year, from USD 106.53 in 1997 purchasing power parity, to USD 293.79 in 2012 [18]. The difference in expenditure could be due to different approaches in the methodology of calculating costs, duration of illness for which the cost was estimated, severity, and type of the disease. The time frame of the studies conducted, periodic variations due to inflation, and healthcare utilization at different levels of the health system limit the extension of comparison. However, considering the average annual per capita income of households as Rs 44,901, according to the Longitudinal Aging Study in India, 2017-18, the direct costs calculated in our study are nearly one-fifth of the annual income [19]. This shows that healthcare costs in this context are still quite unaffordable.

However, the findings of the present study and other studies over India unequivocally underline the fact that costs of diabetes care are exorbitant with a higher economic burden, particularly on the poor. Health system interventions are needed to tackle this keeping in view the increase in the absolute number of diabetics and long-term complications. Universal health coverage with increased accessibility and affordable care can be helpful in ameliorating this problem. Additionally, preventive approaches need to be stressed upon for adequate control of the disease and long-term complications. Screening, early disease detection, and initiation of treatment will be some of the most cost-effective strategies for countering diabetes [20-22]. Also, there should be an emphasis on the aspects of prevention and patient health education, along with a focus on health literacy.

India being a resource-limited setting needs efficient utilization of resources along with community-based preventive approaches to manage the disease effectively. The primary care system, particularly primary health centers, can be the major player in reducing the escalating costs of diabetes care by providing adequate access to drugs, investigation, and laboratory facilities. Strengthening the public health system with a focus on primary care and the expansion of the health coverage blanket are the potential solutions subsequent to the diabetes epidemic.

The present study has some limitations. Patient-reported data are likely to be influenced by recall bias. Efforts were made on the part of interviewers to minimize the errors by adhering to a standardized pattern of interviews and using appropriate probes to maintain data accuracy. Inpatient costs, if any, were not included in the study though very few of the patients reported having been admitted in the last year. It also did not take into account the health system perspective of costs. The compliance and control status of the study participants was also not assessed. However, our study has generalizability in the context of ambulatory care, where patients are buying medicines mostly from private chemists or some subsidized public sector undertaking. In the context of public health facilities where the medications are available free of cost, the costs might be lower. Nevertheless, to our knowledge, this is the first cost of illness study from eastern India on diabetes. It would help in developing comprehensive knowledge on economic implications of the disease and would also help from a policy formulation point of view.

## Conclusions

The study outlines that cost of diabetes treatment is a significant financial burden in a developing country like India, even in an ambulatory framework. Given the resource constraints in Indian health settings, the public health system needs to be adequately strengthened by policymakers to address the ever-increasing number of diabetics and long-standing complications. Increased availability and access to essential anti-diabetic drugs will lead to better management and reduce out-of-pocket expenditure in public sector facilities.
